# Pharmacological modulation of histone demethylase activity by a small molecule isolated from subcritical water extracts of *Sasa senanensis* leaves prolongs the lifespan of *Drosophila melanogaster*

**DOI:** 10.1186/1472-6882-12-101

**Published:** 2012-07-18

**Authors:** Yuzo Nakagawa-Yagi, Yukiko Sato, Emi Matsumoto, Shin-ichi Nakatsuka, Tsuyoshi Sakaki, Yukiko Muramatsu, Takaaki Hara, Toshiro Aigaki

**Affiliations:** 1 Hakuju Institute for Health Science Co. Ltd, 37-5, Tomigaya 1-chome, Shibuya-ku, Tokyo, 151-0063, Japan; 2 Department of Biological Sciences, Tokyo Metropolitan University, 1-1 Minami-osawa, Hachioji, Tokyo, 192-0397, Japan; 3 Nagara Science Co. Ltd, 479-15 Nagase, Oritate, Gifu, 501-1132, Japan; 4 National Institute of Advanced Industrial Science and Technology (AIST) Kyushu, 807-1 Shuku-machi, Tosu, Saga, 841-0052, Japan

**Keywords:** *Drosophila*, Histone demethylase, Lifespan extension, Traditional Japanese medicine

## Abstract

**Background:**

Extracts of *Sasa senanensis* Rehder are used in traditional Japanese medicine; however, little is known about the underlying mechanisms of their potential health benefits.

**Methods:**

*S. senanensis* leaves were extracted with subcritical water. An active small-molecule was isolated using reversed-phase high-performance liquid chromatography (HPLC), and identified as 3,4-dihydroxybenzaldehyde (protocatechuic aldehyde or PA). The effects of PA on the activity of histone demethylase, the *Drosophila melanogaster* lifespan and gene expression in *Drosophila* S2 cells were investigated.

**Results:**

PA inhibited the activity of Jumonji domain-containing protein 2A (JMJD2A) histone demethylase in a dose-dependent manner with a half-maximal inhibitory concentration (IC_50_) of 11.6 μM. However, there was no effect on lysine-specific demethylase 1 (LSD1), histone deacetylase 1 (HDAC1) or HDAC8. PA significantly extended the lifespan of female, but not male, *Drosophila*. In *Drosophila* S2 cells, the eukaryotic translation initiation factor 4E binding protein (4E-BP) was up-regulated by PA exposure.

**Conclusions:**

Our findings provide insight into the possible relationship between the pharmacological modulation of histone demethylation and lifespan extension by PA; they might also be important in the development of alternative therapies for age-related disorders.

## Background

Polyphenols are widely found in natural products [1], and have generated much interest because of the health benefits derived from their antioxidant activities as free-radical scavengers [2]. Leaves from the bamboo genus *Sasa* are known to have anti-microbial, anti-allergic and anti-invasion properties, and have been used in Japanese traditional medicine for treating hypercholesterolemia, obesity and cancer [3-5]. Several studies have identified glycosyl flavones from *Sasa veitchii*, *Sasa borealis* and *Sasa kurilensis*[6-8], but the bioactive molecule of *Sasa senanensis* has not yet been clarified.

The fruit fly *Drosophila melanogaster* has been widely used in aging research, because of the extensive knowledge of its biological pathways, which are conserved in other organisms including humans. A wide variety of mutants and transgenic strains, including inducible RNA interference (RNAi) lines, might sensitise the detection of the biological activity of compounds and lead to the identification of targets *in vivo*[9]. For example, wine-derived resveratrol (*trans*-3,5,4′-trihydroxystilbene) was shown to extend the *Drosophila* lifespan, concomitantly with stimulation of Sir2 activation [10].

The current study isolated a small-molecule antioxidant with superoxide anion radical scavenging activities (SOSA) from subcritical water extracts of *S. senanensis* leaves, and identified the small molecule as 3,4-dihydroxybenzaldehyde (protocatechuic aldehyde or PA). We screened the biological activity of PA in the current context, and examined its effects on the lifespan of *Drosophila*.

## Methods

### Purification and identification of PA

*S. senanensis* plants were collected from Mount Daisetsu in Hokkaido, Japan. The leaves were finely ground to pass through a 100-mesh screen, then used for subcritical extraction with water at 280°C and 10 MPa in a previously described home-built apparatus [11]. The subcritical water extract (1,208 mg) was applied to an octadecylsilane (ODS) column (NX-ODS-9-120A, 28 mm i.d. × 250 mm; Nagara Science, Gifu, Japan), and 10 fractions were eluted stepwise with methanol (MeOH)/hydrogen peroxide (H_2_O_2_;17:83) or with MeOH using an HPLC system equipped with a PU-2087 preparative pump (JASCO, Tokyo, Japan).

SOSA was determined by a spin-trapping method using an electron-spin resonance (ESR) spectrometer (JES-FR80; JEOL, Tokyo, Japan), as described previously [12]. The candidate fraction (fraction 4) was further fractionated by the ODS column (NX-ODS-9-120A;20 mm i.d. × 250 mm; Nagara Science) with an eluting solvent comprising MeOH/acetonitrile/acetic acid/H_2_O (4:3:1:92). The molecular formula of fraction 4-II was identified by EI-MS (JMS-700/GI; JEOL), ^1^H-NMR (UNITY INOVA500, Varian, CA) and ^13^C-NMR (JNM ECA-500, JEOL). The structure was identified with the aid of the AIST SDBS website (http://riod01.ibase.aist.go.jp/sdbs/).

### Adipocyte differentiation assay

Human pre-adipocytes (Zen-Bio, Inc., Research Triangle Park, NC) obtained from abdominal fat-reduction surgeries were cultured up to 80% confluency in preadipocyte growth medium (Zen-Bio, Inc.). Differentiation was induced by treating the cells with differentiation medium containing insulin, dexamethasone, IBMX and PPARγ agonist (Zen-Bio, Inc.). Subsequently the cells were maintained in adipocyte medium, which is identical to differentiation medium but lacks IBMX and PPARγ agonist (Zen-Bio, Inc.) for 7 days. Triglyceride accumulation was measured by the Infinity^TM^ triglyceride reagent kit (Sigma-Aldrich, St. Louis, MO).

### Histone demethylase activity assay

The histone demethylase activity of JMJD2A-C was assessed using the fluorogenic JMJD assay kit (BPS Bioscience, San Diego, CA) according to the manufacturer’s instructions. Inhibition assays were carried out in 384-well plates. The assay volume was 10 μl, and contained biotinylated histone H3 peptide substrate, demethylase enzyme and varying concentrations of the test compound in assay buffer. PA or apocynin was dissolved in dimethyl sulphoxide (DMSO). The formation of the fluorescent product was measured using a SpectraMax M2 plate reader (Molecular Devices, Sunnyvale, CA). The excitation and emission wavelength were 360 and 450 nm, respectively. The concentrations of PA required to inhibit 50% of the demethylase activity of a JMJD2 isoform were calculated by regression analysis using SigmaPlot software (Systat Software, Inc., San Jose, CA).

### Molecular modelling

Docking and subsequent scoring were performed using Sybyl-X1.3 software (Tripos Inc., St. Louis, MO).

### *Drosophila* and media

Unless otherwise stated, the *Drosophila* were reared on standard medium (9% cornmeal, 10% glucose, 4% dry yeast, 0.8% agar, 0.3% propionic acid and 0.1% *p*-hydroxybutylbenzoate) at 25°C. PA was dissolved in ethanol, and added to the standard medium or glucose-based medium (10% glucose, 2% agar and 0.3% propionic acid) before it solidified. Medium containing ethanol alone was used as a control. The yw (y1w67c23) strain of *Drosophila* was used in all experiments.

### Lifespan assay and viability

Lifespan analysis was performed as described previously [13]. During development, the *Drosophila* were reared on standard medium containing PA or ethanol as a control.

Newly eclosed *Drosophila* were kept in plastic chambers containing the glucose-based medium supplemented with either PA or ethanol (control). Five males or females were placed in the chamber, and 120 *Drosophila* were used for each assay. *Drosophila* were transferred to new chambers containing fresh medium every 2–3 days, and the number living. Twenty *Drosophila* aged 5–10 days were placed on standard medium and allowed to mate for 1 h, after which they were transferred to culture vials containing standard medium plus various concentrations of PA and allowed to lay eggs for 2 h. The culture vials were kept at 25°C. Viability was calculated by counting the number of eggs laid on the media and the number of eclosed *Drosophila* in each vial. Three culture vials were used for each concentration of PA.

### Affymetrix GeneChip microarray

*Drosophila*-derived S2 cells were cultured in Schneider’s *Drosophila* medium (Invitrogen, Life Technologies, Carlsbad, CA) supplemented with insulin (10 μg/mL; Nakarai Tesque, Kyoto, Japan) and 10% fetal bovine serum (FBS; HyClone Laboratories, South Logan, UT). Briefly, 10^6^ cells/well were seeded in six-well multiwall plates. RNA isolated from each sample was processed and hybridized to an Affymetrix GeneChip Drosophila genome 2.0 array according to the protocols described in the GeneChip Expression Analysis Technical Manual (Affymetrix, Santa Clara, CA). Raw data was submitted to National Center for Biotechnology Information (NCBI) Gene Expression Omnibus (GEO) database (http://www.ncbi.nlm.nih.gov/geo/, platform accession number GSE37701).

### Quantitative (q) RT-PCR

Total RNA from two mycelia fragments was isolated using the RNeasy Plant Mini Kit (Qiagen, Valencia, CA). The total RNA (1 μg) was reverse transcribed using Rever Tra Ace (Toyobo, Osaka, Japan). The primers were as follows: *4E-BP*-F, CCA AAC TCC GCC GTC CAA CGT GCC C; *4E-BP*-R, ACT GTT CCT GGT CCT CAA TCT TCA G; *ferrochelatase*-F, CCT GAC AAA CGT TGT GGC AGA CCA C; *ferrochelatase*-R, TGG CGG TAC CAG CTT TTG CTC TCC C. *GAPDH2*-F, GCG GTA GAA TGG GGT GAG AC and *GAPDH2*-R, TGA AGA GCG AAA ACA GTA GC. All PCR reactions were carried out using SYBR Premix EX Tag (Takara Bio, Otsu, Japan). Amplification and detection was performed using the following program: 95°C (10 sec) and 60°C (1 min) for 50 cycles. Fold induction values were calculated according to the equation 2^ΔΔ^Ct, indicating the differences in cycle threshold numbers between the target gene and *GAPDH2*, and ^ΔΔ^Ct represents the relative values in the differences between control and treatments.

### Chemicals

3,4-dihydroxybenzaldehyde as a synthetic standard compound and resveratrol were purchased from Kanto Chemical (Tokyo, Japan). 2,4-pyridinedicarboxylic acid (2,4-PDCA) and apocynin (4-hydroxy-3-methoxyacetophenone) were purchased from Sigma-Aldrich Chemie GmbH (Steinheim, Germany).

### Statistical analysis

Statistical analysis was performed using R version 2.10.1 (http://cran.r-project. org/). The log-rank (Mantel-Cox) test was used to determine differences in survival curves and mean lifespan. Analysis of variance (ANOVA) and Student’s *t*-test were used to compare viability data between groups. Values of *p*<0.05 were considered statistically significant.

## Results

### Isolation and identification of PA from subcritical water extracts of *S. Senanensis* leaves

**Figure 1:**
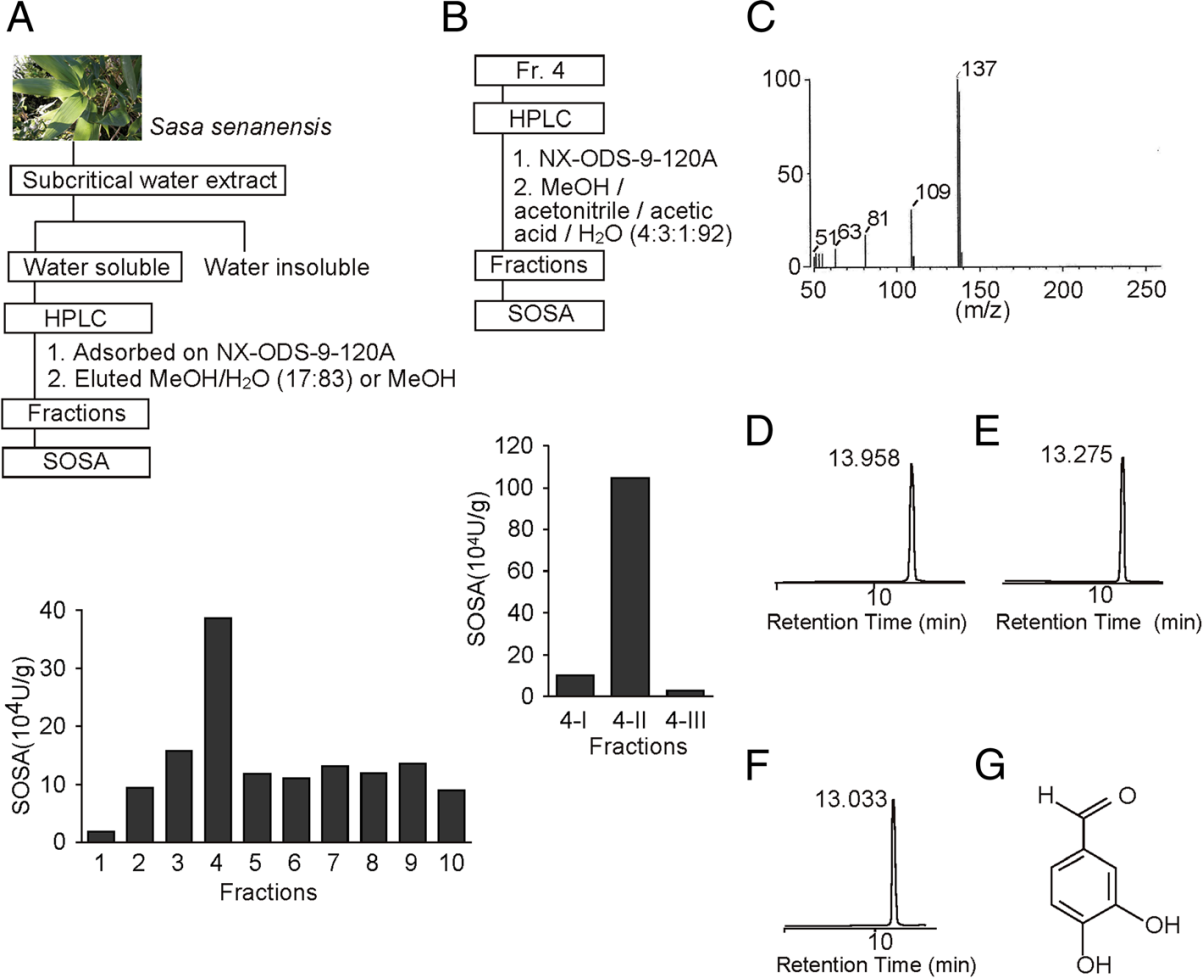
**Purification and identification of PA.** (**A**) Purification of antioxidative compounds from *S. senanensis* leaves. Subcritical water extracts were prepared from the leaves and fractionated by HPLC with MeOH/H_2_O (17:83). The SOSA was assessed for each fraction, and the highest value detected in fraction 4. (**B**) Purification of the compound by HPLC. Fraction 4 was further fractionated, and fraction 4-II found to contain the highest SOSA. (**C**) EI-MS spectra data of purified PA, which was identified using the SDBS. (**D**–**F**) HPLC chromatograms of fraction 4-II (**D**), synthetic PA (**E**), and a mixture of fraction 4-II and synthetic PA (**F**), in which the two compounds were co-eluted as a single peak. (**G**) Chemical structure of PA.

To identify the active small-molecule present in *S. senanensis* leaves, we prepared subcritical water extracts at 280°C and 10 MPa, and fractionated them by reversed-phase high-performance liquid chromatography (HPLC). Fraction 4 was identified as having antioxidant activity, as its SOSA measurement was relatively high (Figure 1A); it was therefore further fractionated by HPLC to obtain fraction 4-II, which had the highest activity of all the fractions (Figure 1B). Lyophilisation of fraction 4-II yielded a light-yellow powder and electron ionization-mass spectrometry (EI-MS; m/z 138[M]^+^; Figure 1C) and ^13^C- nuclear magnetic resonance (NMR) showed its molecular formula to be C_7_H_6_O_3_. ^1^H-NMR spectral data indicated the presence of a 1,3,4-trisubstituted benzene ring at δ 7.3 (2H) and δ 6.9 (1H), whereas δ 9.7 showed a singlet signal of an aldehyde group.

Using these data, we searched the National Institute of Advanced Industrial Science and Technology (AIST) Spectral Database for Organic Compounds (SDBS; see Methods), which suggested PA as a candidate substance. To confirm the identity of this molecule, we compared the HPLC retention time between fraction 4-II and synthetic PA. As shown in Figure 1D–F, the substance contained in this peak co-eluted with synthetic PA, suggesting that PA was indeed the major compound with SOSA in the subcritical-water extracts of *S. senanensis* leaves (Figure 1G).

### Effect of PA on adipocyte differentiation

**Figure 2:**
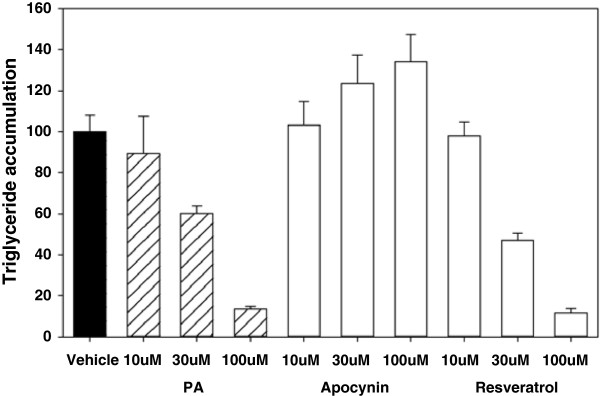
**Effect of PA on adipocyte differentiation.** Inhibition of human subcutaneous preadipocyte differentiation by PA. Differentiation was determined by the amount of lipid accumulation measured by total triglyceride. Data are expressed as a percentage of the control value. Values are mean ± standard error of the mean (SEM; n = 3).

Resveratrol is not only an NAD^+^-dependent deacetylase activator but also inhibits lipid-droplet accumulation in adipocytes [14]. We thus examined the effect of PA on human subcutaneous preadipocyte differentiation into adipocytes. As shown in Figure 2, PA caused a decrease in the amount of triglyceride in the adipocyte differentiation of human preadipocytes induced by insulin, isobutylmethylxanthine (IBMX), peroxisome proliferator-activated receptor γ (PPARγ) agonist and dexamethasone. This inhibitory effect was dose-dependent for PA concentrations ranging from 10 to 100 μM, and the half-maximal inhibitory concentration (IC_50_) for differentiation was about 30 μM. Similar results were obtained using resveratrol instead of PA. Under these conditions, the NADPH oxidase inhibitor apocynin was less effective than PA in inhibiting adipocyte differentiation.

### PA inhibits histone demethylase activity

**Figure 3:**
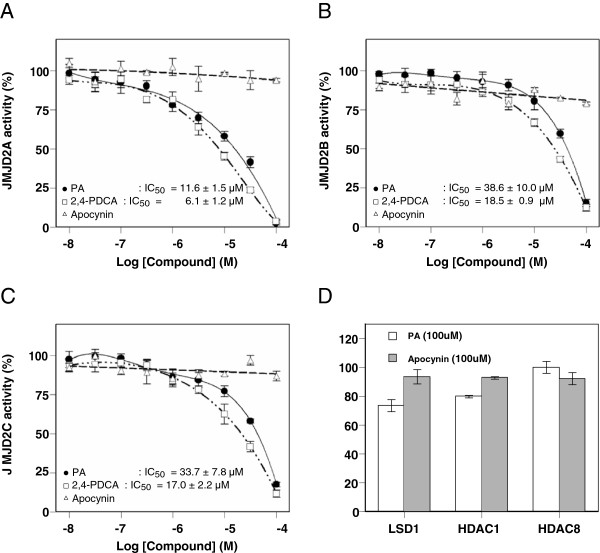
**Effects of PA on the activities of histone demethylases.** (**A**) Effects of PA, apocynin or 2,4-PDCA on histone demethylase JMJD2A activity. IC_50_ values are mean ± SEM of three separate experiments. (**B**) Effects of PA, apocynin or 2,4-PDCA on histone demethylase JMJD2B activity. IC_50_ values are mean ± SEM of three separate experiments. (**C**) Effects of PA, apocynin or 2,4-PDCA on histone demethylase JMJD2C activity. IC_50_ values are mean ± SEM of three separate experiments. (**D**) Effects of PA or apocynin on the activities of lysine-specific demethylase LSD1, histone deacetylase HDAC1 and HDAC8. Values are mean ± SEM (n = 4). Data are expressed as a percentage of the control value in each experiment.

3,4-dihydroxybenzoate, which has a similar chemical structure to PA, is capable of inhibiting the 2-oxoglutarate binding sites of prolyl 4-hydroxylase [15]; we thus tested the effects of PA on 2-oxoglutarate-dependent oxygenases in histone demethylation. A role for histone demethylation has previously been established during adipocyte differentiation [16,17]. As shown in Figure 3A3C, PA decreased the activities of Jumonji domain-containing protein 2A (JMJD2A), JMJD2B and JMJD2C, and this inhibitory effect was dose-dependent for PA concentrations. The IC_50_ values were 11.6 ± 1.5, 38.6 ± 10.0 and 33.7 ± 7.8 μM for JMJD2A, JMJD2B and JMJD2C, respectively.

Regarding JMJD2A activity, PA was 1.9-fold less potent than the JMJD2 inhibitor 2,4-PDCA. Under these conditions, apocynin had no effect on the activities of JMJD2A, JMJD2B and JMJD2C. To examine whether other types of histone demethylase could be similarly inhibited by PA, we tested the effect of PA on lysine-specific demethylase 1 (LSD1); however, 100 μM PA had no effect on LSD1 activity (Figure 3D). There was also no effect of PA on the activities of histone deacetylase 1 (HDAC1) and HDAC8 as examples of non-demethylase activity (Figure 3D).

**Figure 4:**
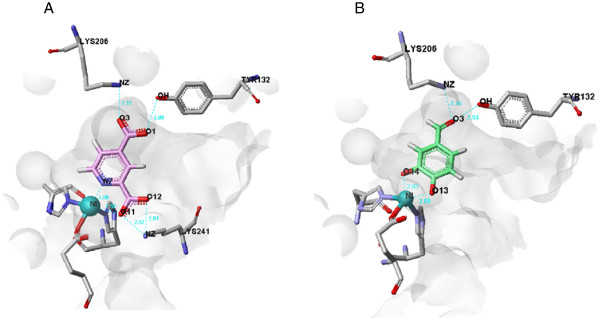
**View of the conformation of PA docked in the JMJD2A active site.** (**A**) Binding mode of 2,4-PDCA in JMJD2A. (**B**) Binding mode of PA in JMJD2A.

The crystal structures of complexes with inhibitors have been reported for the histone demethylase JMJD2A [18]; we therefore performed a binding mode study of PA in the active site of JMJD2A using Sybyl-X1.3 software (Figure 4). The results indicated that PA would bind to JMJD2A.

### PA extends the lifespan of *Drosophila in vivo*

**Figure 5:**
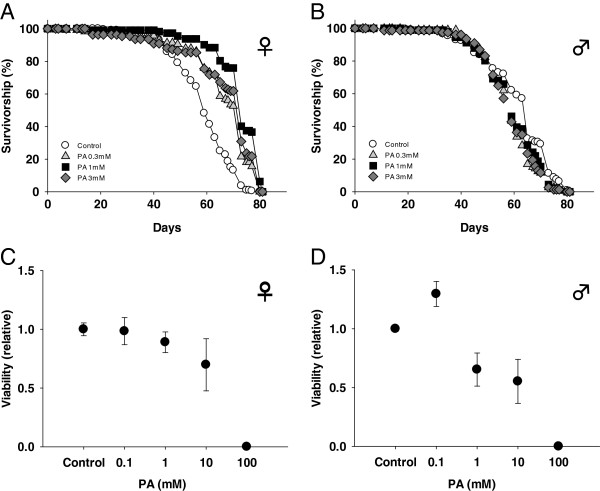
**Effects of PA on the lifespan and egg-to-adult viability of *Drosophila.*** (**A**) Survival curves of females kept on media containing PA. The mean lifespan of female *Drosophila* kept on 0.3, 1 and 3 mM PA was increased by 13%, 23% and 13%, respectively (*p*<0.001; log-rank test). (**B**) The survival curves of males revealed no effects of PA. (**C**) The viability of females was not affected at concentrations of up to 10 mM. (**D**) The viability of males was reduced by PA, even at 1 mM. Larval development was arrested at 100 mM in both genders (*p*<0.05, *t*-test).

We next examined the effects of PA on the lifespan of adult *Drosophila* kept under normal culture conditions. The mean lifespan of female *Drosophila* fed 0.3, 1 and 3 mM PA was increased by 13, 23 and 13%, respectively (p<0.001 for all; Figure 5A). However, no significant difference in lifespan was observed in male *Drosophila* (Figure 5B). To assess the toxicity of PA *in vivo*, we examined its effects on the egg-to-adult viability of *Drosophila* reared on media containing different concentrations of PA (Figure 5C– 5D). This revealed a gender difference in PA toxicity, with males being more sensitive and showing a slightly reduced viability during larval development at 1 and 10 mM PA. Larval development of both males and females was arrested at 100 mM PA.

### Gene expression analysis of *Drosophila* S2 cells

**Figure 6:**
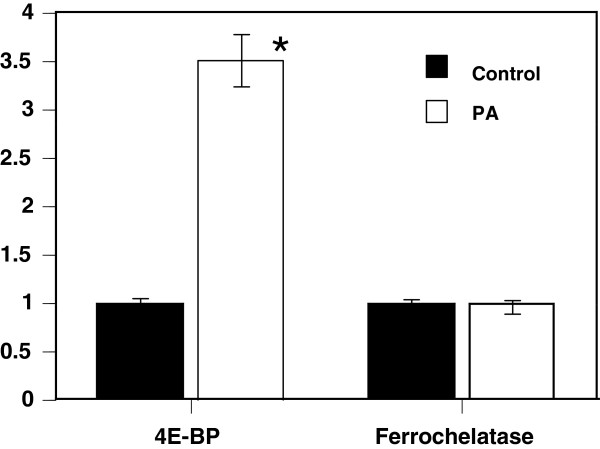
**Effect of PA on gene expression in *Drosophila *S2 cells.** qRT-PCR analysis of 4E-BP or ferrochelatase mRNA in *Drosophila* S2 cells treated with 100 μM PA for 2 h. Glyceraldehyde 3-phosphate dehydrogenase (GAPDH) is included as an internal control. Results are mean ± SEM (n = 5). *p<0.01 compared with vehicle (0.1% DMSO).

An Affymetrix GeneChip *Drosophila* genome 2.0 array was used to study the effect of PA on gene expression. As shown in Table 1, the addition of 100 μM PA to *Drosophila* S2 cells significantly affected the expression of 52 genes, with 29 being up-regulated and 23 being down-regulated. Since PA-induced up-regulation of the eukaryotic translation initiation factor 4E binding protein (4E-BP) was observed in microarray analysis, we next confirmed the effect of PA on 4E-BP at the messenger RNA level by quantitative reverse transcription polymerase chain reaction (qRT-PCR) analysis. As shown in Figure 6, treatment with PA produced about a 3.5-fold increase in qRT-PCR analysis. In contrast to 4E-BP, ferrochelatase as a negative control was quite inactive.

**Table 1:** **Expression of selected genes exhibiting > 1.5-fold or < 0.67-fold changes in *Drosophila *S2 cells after exposure to 100 μM PA for 2h**

**Accession number**	**Gene symbol**	**Gene description**	**Fold change**
Up-regulated genes			
NM_001103559	CG34330	_	10.0
NM_001038793	CG	_	3.78
NM_057233	*impL3*	lactic DH	3.78
NM_141268	*Hph*	fatiga	2.99
NM_001103964	*CG9815*	_	2.93
NM_140239	*CG11652*	_	2.82
NM_080713	*fok*	fledgling of Klp38B	2.80
NM_057947	*Thor*	eukaryotic initiation factor 4E-binding protein	2.26
NM_001144611	*bnl*	fibroblast growth factor	2.24
NM_058032	*Fpps*	Famesyl diphosphate synthase	2.15
Nm_079270	*Hsp67Bc*	Gene 3	2.07
NM_137458	*Dgp-1*	Dgp-1	2.02
NM_080173	GstD2	Glutathione S transferase D2	1.81
NM_170034	*Gclm*	Glutathione-cysteine ligase modifier submit	1.81
NM_138269	*cue*	cueball	1.68
NM_135115	*CG14005*	_	1.67
NM_001032272	*CG3785///CG33786*	_///_	1.65
NM_001170286	_	_	1.64
NM_001031943	*Hsp22///Hsp67Bb*	heat shock protein hsp22///Gene 2	1.63
NM_143700	*CG17724///seq*	_///sequoia	1.63
NM_141334	*CG10979*	_	1.61
NM_134584	*CG32512*	_	1.61
NM_001043271	*SNF4Agamma*	Lochrig	1.61
NM_140199	*scyl*	scylla	1.59
NM_144004	*CG3348*	_	1.58
NM_206410	*asaragine-synthetase*	asparagine synthetase	1.57
NM_135328	*Spn28D*	Dm-serpin-28D	1.57
NM_001144540	*CG2017*	GP-1 related	1.55
NM_137485	*GstE7*	Glutathione S transferase E7	1.52
Down-regulated genes			
NM_138240	*CG9194*	_	0.55
NM_143815	*Invadolysin*	invadolysin	0.59
NM_140792	*MYPT-75D*	MYPT-75D	0.59
NM_136764	*wde*	Windei	0.60
NM_057696	*Ret*	Ret oncogene	0.60
NM_079534	*alpha-Est 9*	fragment J	0.62
NM_130578	*CG32809*	_	0.62
NM_079937	*sls*	sallimus	0.62
NM_001014750	*mnb*	minibrain	0.62
NM_079578	*jumu*	Domina	0.63
NM_080370	*ltd*	lightoid	0.63
NM_001103650	*Btk29A*	Btk family kinase at 29A	0.63
NM_168566	*CG32135*	_	0.63
NM_206332	*Muc68Ca*	Muc in 68Ca	0.64
NM_001031884	*CG33691///CG33692*	///	0.64
NM_176545	*unk*	unkempt	0.65
NM_168366	*CG42268*	CG32044	0.65
NM_001169959	*Sema-5c*	Semaphorin 5C	0.65
NM_078959	*Prx2540-2*	peroxiredoxin 2540	0.66
NM_001104450	*jigr1*	CG17383	0.66
NM_001169949	*Pi3K68D*	dPI 3-kinase	0.66
NM_001169228	*fs(1)h*	rancor	0.66
NM_078607	*dah*	Apodystrophin	0.67
NM_137485	*GstE7*	Glutathione S transferase E7	1.52

Fold changes relative to vehicle-treated cells. Genes were differentially transcribed at *p*<0.05 (*t*-test).

## Discussion

Histone demethylation has been suggested to play an important role in the lifespan of model organisms [19,20]. However, much of the evidence for this came from manipulations made using RNAi-mediated knockdown. Here we report additional evidence in favour of the inhibitory effects of the histone demethylase JMJD2 family by the small molecule PA. Previously, PA was known as a polyphenol that is naturally found in the fruiting bodies of *Phellinus linteus*, *Ganoderma applanatum* and *Ranunculus sieboldii*, the roots of *Salvia miltiorrhiza*, the leaves of *Vitis vinifera*, and grape and barley seeds [21-27]. It was shown to inhibit the activities of tyrosinase, herpes simplex virus type-1 replication, tumour necrosis factor (TNF)-α-induced cell-surface expression of vascular adhesion molecule-1, aldose reductase, phosphatidylinositol kinase and advanced glycation end product-bovine serum albumin (BSA) formation [28-31].

Several studies reported on the use of pharmacological manipulation with transcription factors and nucleosomal histone modification to inhibit adipocyte differentiation [16,17]. To gain further knowledge of relative efficacy, the inhibitory effects of PA were compared with a well-known JMJD2 family inhibitor, 2,4-PDCA. PA was approximately 2-fold less potent than 2,4-PDCA in affecting JMJD2A activity. Moreover, inhibitory changes by PA were sensitive to the JMJD2 family but insensitive to LSD1, suggesting that PA might involve 2-oxoglutarate oxygenase from the Jumonji domain-containing family, but not amine oxidase in lysyl demethylase. To better understand the property of PA, it is important to clarify the specificity of PA against different classes of histone demethylases. As 2,4-PDCA and the collagen proline hydroxylase inhibitor 3,4-dihydroxybenzoate bind to the 2-oxoglutarate binding site of prolyl 4-hydroxylase, inhibition of the 2-oxoglutarate co-substrate in the JMJD2 family is a potential mechanism [12,32]. Further studies are needed to elucidate the identity of the demethylation site that may be affected by PA. Crystallographic analyses of JMJD2A in complex with 2,4-PDCA have shown that it binds in a similar manner to the 2-oxoglutarate co-substrate [15,33]. In the present study, molecular computational modelling analysis showed that PA can bind to JMJD2A at the active site, in a similar manner to 2,4-PDCA.

In humans, about 30 JmjC proteins have been identified and grouped into eight distinct subfamilies: JHDM1, JHDM2, JMJD2, PHF2, PHF8, Jumonji(A + T)-rich interactive domain (JARID), ubiquitously transcribed tetratricopeptide repeat X/Y-linked (UTX/UTY) and JmjC-domain [34]. Han and colleagues observed that RNAi of the utx-1 gene extends the mean lifespan of *Caenorhabditis elegans* by about 30% [19]. By contrast, Li et al. recently reported that histone demethylase-inactive Lid flies are short lived [20], whereas another study showed that disruption of Dmel/Kdm4A, a homologue of the human JMJD2 family, reduces male-specific longevity [35]. Lifespan is highly sensitive to genetic background and environmental conditions. Therefore, it is possible that the physiological situation is different between Kdm4A mutant flies and those treated with PA. Lifespan of Kdm4A mutant male was shorter than wild-type, while that of mutant females was unchanged. In contrast, PA extended the lifespan of female, but not male. Interestingly, its toxicity was more obvious in males than in females. Therefore, there is a consistency of sex difference: females were more tolerant to the reduced activity of Kdm4A compared to males. PA might have additional functions including inhibitory activity against other KDM4/JMJD2 demethylases. Further studies are needed to clarify the mechanism of lifespan extension by PA.

In the present study, *Drosophila* lifespan extension was specific to female individuals. Gender-specific lifespan extension has been reported in several *Drosophila* mutants, including the insulin substrate *chico*, kelch-like ECT-associated protein 1 (*keap1*), *p53* and puckered (*puc*) mutants [36-39].

Another goal of the present study was to gain insights into the genetic components affected by PA through a large-scale analysis of gene expression. In *Drosophila* S2 cells, 4E-BP was up-regulated in response to PA. 4E-BP has been reported to play an important role in lifespan extension following dietary restriction in *Drosophila*[40]. Moreover, Demontis et al. recently reported that key roles of FOXO/4E-BP signaling are to preserve muscle function and extend the lifespan of *Drosophila*[41]. Thus, extension of *Drosophila* lifespan by PA might involve, at least in part, the 4E-BP signal cascade. It is unclear at present whether PA induces 4E-BP via the insulin receptor/4E-BP pathway. It is reasonable hypothesis that PA might extend the lifespan through downregulating the insulin/IGF signaling pathway. Further studies are needed to elucidate whether the intracellular 4E-BP-dependent signaling pathway induced by PA might affect the lifespan extension of *Drosophila*.

## Conclusions

PA from subcritical water extracts of *S. senanensis* leaves showed notable inhibitory effect on the histone demethylase JMJD2A. Moreover, PA significantly extended the lifespan of female *Drosophila*. In addition to identifying PA as a histone demethylase JMJD2 family inhibitor, we suggest a model for how JMJD2 enzymes might be involved in lifespan extension, and propose PA as a target for anti-aging.

## Abbreviations

4E-BP: Eukaryotic translation initiation factor 4E binding protein; EI-MS: Electron ionization-mass spectrometry; ESR: Electron-spin resonance; GAPDH: Glyceraldehyde 3-phosphate dehydrogenase; HDAC: Histone deacetylase; HPLC: High-performance liquid chromatography; IBMX: 3-isobutyl-1-methylxanthin; JMJD: Jumomji domain-containing protein; LSD: Lysine-specific demethylase; NMR: Nuclear magnetic resonance; PA: Protocatechuic aldehyde; qRT-PCR: Quantitative reverse transcription polymerase chain reaction; SDBS: Spectral database for organic compounds; PPAR: Peroxisome proliferator-activated receptor; Sir2: Silent information regulator 2; SOSA: Superoxide-anion-radical scavenging activity.

## Competing interests

YN-Y, YM and TH are employees of Hakuju Life Science Co. Ltd., and EM and SN are employees of Nagara Science Co. Ltd. All other authors have no competing interests.

## Authors’ contributions

YN-Y and YS contributed equally to this work. YN-Y, YS and TA designed and supervised the research, and wrote the manuscript. YS performed biological experiments. YN-Y, YM and TH performed molecular pharmacological experiments. EM and SN performed phytochemical experiments. TS performed subcritical extractions. All authors read and approved the final manuscript.

## Pre-publication history

The pre-publication history for this paper can be accessed here:

http://www.biomedcentral.com/1472-6882/12/101/prepub
